# Ruptured Ovarian Cystic Teratoma: A Rare Diagnosis, Easily to Be Confused with Peritoneal Carcinomatosis

**DOI:** 10.3390/medicina60030460

**Published:** 2024-03-10

**Authors:** Dan Costachescu, Alexandru Catalin Motofelea, Daniel Malita, Florica Birsasteanu, Ioana Ionita, Nadica Motofelea, Cristina Ana-Maria Jura, Ioana-Flavia Bacila, Mihai Bacila, Sorin Motoi

**Affiliations:** 1 Radiology Department, University of Medicine and Pharmacy “Victor Babes” Timisoara, 300041 Timisoara, Romania; costachescu.dan@umft.ro (D.C.); malita.daniel@umft.ro (D.M.); fbirsasteanu@umft.ro (F.B.); motoi.sorin@umft.ro (S.M.); 2 Radiology Department, City Emergency Hospital Timisoara, 300202 Timisoara, Romania; 3 Department of Internal Medicine, “Victor Babes” University of Medicine and Pharmacy Timisoara, Eftimie Murgu Square 2, 300041 Timisoara, Romania; 4 Hematology Department, University of Medicine and Pharmacy “Victor Babes” Timisoara, 300041 Timisoara, Romania; ionita.ioana@umft.ro; 5 Department of Obstetrics and Gynecology, “Victor Babeş” University of Medicine and Pharmacy, Eftimie Murgu Sq. No. 2, 300041 Timisoara, Romania; nadica.motofelea@umft.ro (N.M.); cristina.jura@umft.ro (C.A.-M.J.); ioana.bacila@umft.ro (I.-F.B.); 6 Ginecology Department, City Emergency Hospital Timisoara, 300202 Timisoara, Romania; mihaibacila@yahoo.com

**Keywords:** ovarian cystic teratoma, complications, rupture, ultrasound, computed tomography, magnetic resonance imaging

## Abstract

Although ovarian cystic teratoma is the most common ovarian tumor, complications are quite rare. However, it is important to be recognized by the radiologist in order to avoid inaccurately diagnosing them as malignant lesions. This case report describes a 61-year-old postmenopausal woman, who presented to the emergency room with abdominal pain following a minor blunt abdominal trauma. In this context, a CT scan was performed, which showed the presence of round, hypodense masses randomly distributed in the peritoneum, with coexisting ascites in moderate amount; ovarian carcinoma with peritoneal carcinomatosis was suspected. The patient was hospitalized and an MRI of the abdomen and pelvis was recommended for a more detailed lesion characterization. Following this examination, the patient was diagnosed with mature cystic ovarian teratoma complicated by rupture. Surgery was performed, and the outcome was favorable. The cases of ruptured cystic teratomas are rare, and to our knowledge, this is the first occurrence described in literature. Special attention must be paid when confronting with such a case in medical practice, since it can easily misdiagnosed as peritoneal carcinomatosis.

## 1. Introduction

Ovarian teratoma is the most common benign germ cell ovarian tumor (10–20%) [1]. Generally, these lesions are classified in three categories based on the constitutive elements: the first category holds tumors that have layers of detritus/cellular debris, the second characteristically presents a nodular or palm-shaped protrusion, and the last is represented by a fat-fluid level. Over the years, several cases of cystic ovarian teratomas that do not fit into the categories described above have been described, along with the presence of numerous intracystic floating balls, predominantly of fatty consistency. Mature cystic ovarian teratoma can be easily diagnosed with the help of ultrasonography, CT or MRI in situations without complications. The main drawbacks that can occur in the evolution of these lesions are represented by: ovarian torsion (the most common), rupture (1–4%), malignant transformation (1–2%), superinfection, auto-immune hemolytic anemia, hyperthyroidism (more frequently seen in ovarian goiter), carcinoid syndrome or others [1]. Following literature research, this is, to our knowledge, the first case of ruptured mature cystic ovarian teratoma with the presence of floating fatty balls.

## 2. Materials and Methods

This case report describes a ruptured mature cystic ovarian teratoma with the presence of floating fatty balls. For diagnosis and treatment computer tomography (CT) evaluation, magnetic resonance imaging (MRI) and surgery were performed. The CT scan was performed using a Somatom Definition Egde 128 slice scanner( Siemnes, Erlangen, Germany); both pre- and postcontrast scans were done. As for MRI evaluation, it was realized using a 1.5 T MRI scanner (Magnetom Aera, Siemens, Erlangen, Germany); this case also required pre- and post contrast sequences, including, DWI and chemical shift imaging sequences. The surgical treatment included laparatomy with left adnexectomy, peritoneal lavage and lysis of entero-enteric adhesions. A written consent for publication was obtained from the patient.

## 3. Case Presentation

We present the case of a 61-year-old female patient, who has been postmenopausal for about 10 years and referred to the emergency department after a minor abdominal trauma. The patient experienced diffuse abdominal pain which worsened over time. The initial blood count was unremarkable (Table 1).

**Figure 1:**
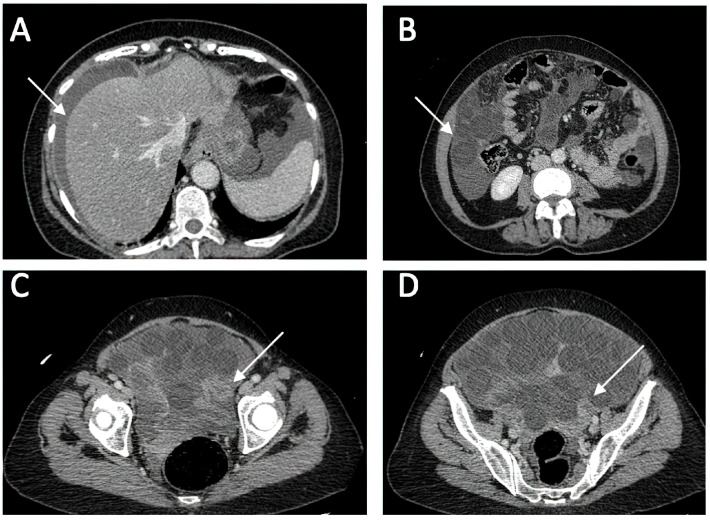
Diagnostic CT Imaging of Abdominal and Pelvic Regions. (**A**) ascites (**B**) multiple round intra-peritoneal fatty density lesions without contrast uptake; (**C**,**D**) serpiginous structure with intense contrast uptake at the level of the left ovary.

**Figure 2:**
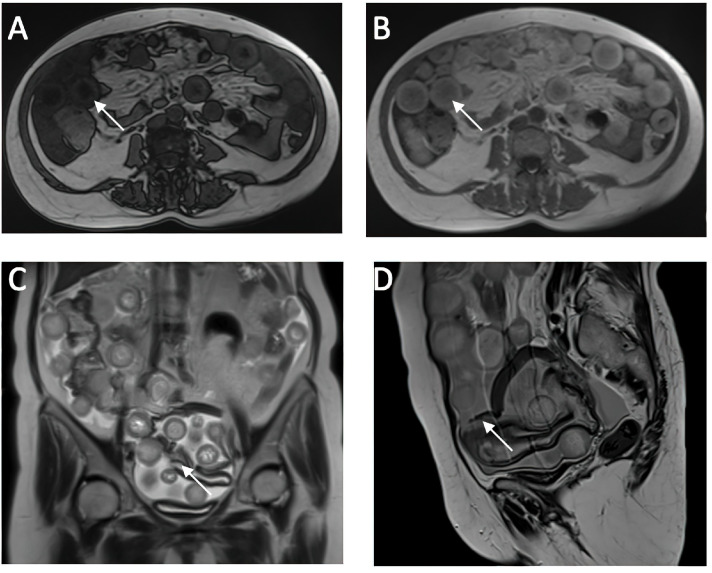
Cross-Sectional MRI Analysis of Abdominal and Pelvic Organs. (**A**,**B**) in-phase and op-phase images showing signal dropout on the op phase, suggesting fat content of the sphere (**C**,**D**) ruptured cyst wall which is hypointense on the T2w sequence.

**Figure 3:**
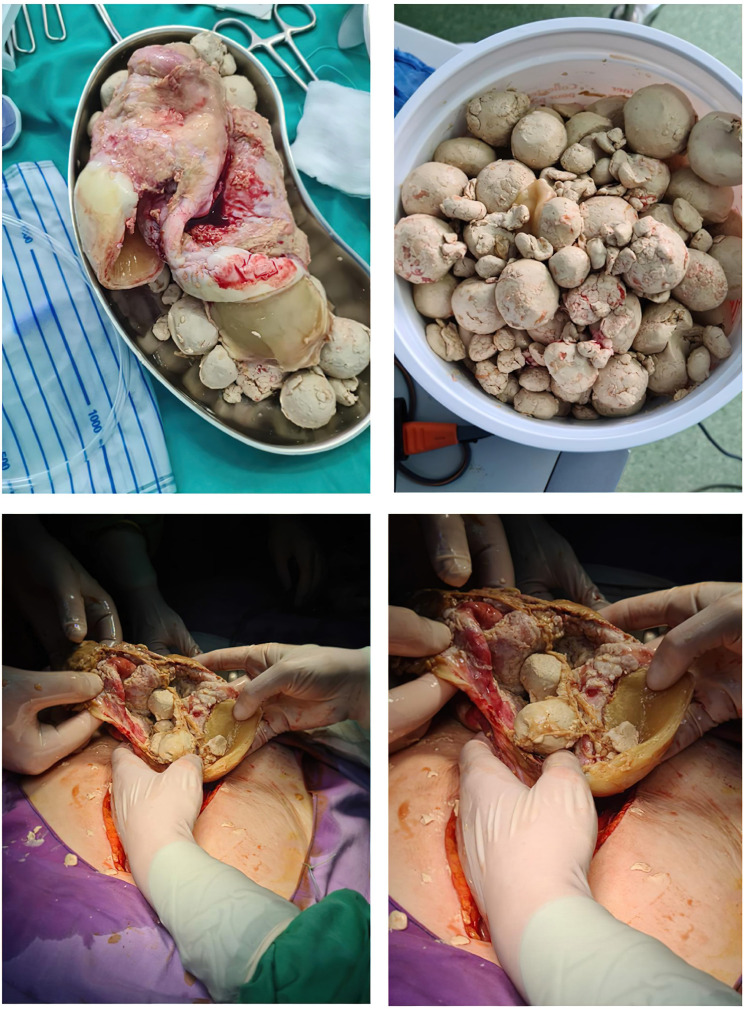
Intraoperative Depiction of Ruptured Ovarian Cyst with ’Floating Balls’ Phenomenon: Surgical Exploration and Findings.

A CT investigation of the abdomen and pelvis was requested and performed. Radiology noted the presence of ascites in moderate amount, together with numerous round nodular lesions of varying sizes (between 1.5–4 cm), with no contrast uptake; the lesions were randomly distributed, with a slight prevalence for the pelvic region. However, a serpiginous lesion with intense contrast uptake was observed within the left ovary (Figure 1), leading the radiologist to falsely raise the suspicion of ovarian carcinoma with peritoneal carcinomatosis. The patient was hospitalized for additional investigations and medical treatment. After several days, an abdominal and pelvic MRI was also performed (Figure 2). Several ‘floating round bodies’ were observed throughout the whole peritoneum; the ‘floating bodies’ showed signal drop on the out-of-phase sequences suggesting fatty components. When analyzing the pelvic images, a ruptured cyst was observed across the right ovary. The diagnosis of ruptured ovarian teratoma was made and the patient underwent surgery. Left adnexectomy with peritoneal lavage and lysis of entero-enteric adhesions was performed. During the laparotomy, the peritoneal cavity was opened revealing a moderate amount of peritoneal fluid. A sample was then collected for cytologic examination. Macroscopically, the uterus had a normal appearance, with unremarkable right adnexa and left fallopian tube. As for the left ovary, a cystic mass with ruptured wall was observed. The cyst contained multiple well defined round lesions with fatty content, some of which were disseminated throughout the peritoneal cavity. To manage postoperative fluid accumulation and monitor for any complications, an abdominal drain was kept in place for 3 days after surgery. The surgical findings are depicted in Figure 3.

Eight days after the surgical intervention, the results of the histopathological examination of the left ovary and peritoneal lavage samples were communicated, leading to the definitive diagnosis: mature cystic ovarian teratoma consisting of various tissues, including epidermal tissue with keratotic squamous epithelium, sebaceous and sweat glands, cartilaginous tissue, brain tissue, connective tissue covered with a cylindrical ciliated epithelium, muscle tissue, and adipose tissue. Additionally, there was an associated chronic non-specific granulomatous inflammation with multinucleated giant cells, with no signs of malignant proliferation. The postoperative evolution was favourable - the patient was discharged with a good general condition. Four months after the surgery, the patient returned to the obstetrics-gynecology clinic for evaluation, and no abnormalities were observed.

## 4. Discussion

Mature cystic ovarian teratoma, the most prevalent benign ovarian neoplasm in young, fertile women, often remains asymptomatic and unilateral (85–90%). Our case involved an incidental discovery of a left adnexal lesion following minor trauma. While typically asymptomatic, teratomas may cause pelvic pain if complicated by torsion or when significantly enlarged. Histologically, these tumors are distinguished by their composition, containing elements from at least two of the three germ layers: ectoderm, mesoderm, and endoderm. Unlike dermoid cysts, which primarily exhibit ectodermal structures, teratomas feature a broader range of tissues, including fat (present in 93% of cases), mature skin, sebaceous glands, hair follicles, sebum bags, and even complex structures like blood vessels, bone fragments, teeth, nails, eyes, cartilage, or thyroid tissue [1]. In relation to the last element, there is a subtype of ovarian teratoma called struma ovarii which is composed entirely or almost entirely of thyroid tissue [2]. In our case, the histopathological examination confirmed the following: epidermal tissue with keratotic squamous epithelium, sebaceous and sweat glands, cartilaginous tissue, brain tissue, connective tissue covered with a ciliated cylindrical epithelium, muscle tissue and adipose tissue. The mature cystic ovarian teratoma can easily be diagnosed using imaging techniques, due to the intra-cystic components which can be detected by ultrasound, CT, or MRI as summarized in Table 2. This benign ovarian neoplasm is divided into three categories based on the composing elements: the first type has layers of debris/cellular remnants, the second holds a nodular protrusion or palm-like projection known as Rokitansky nodule, and the third type contains fat-fluid level. However, there is another category that does not fit into the ones previously mentioned, and is characterized by the presence of floating fatty balls inside the cyst, also known as the “floating balls” sign. This sign is considered pathognomonic for benign ovarian cystic teratomas and is quite common, contrary to expectations [3]. These intracystic spherical lesions were first described on CT by Muramatsu et al. in 1991 [4] and later in 2000 by Otigbah et al. through ultrasound [5]. On ultrasound examination, mature cystic ovarian teratoma presents as multiple hyperechogenic round structures floating in the anechoic fluid of the cyst. In some cases, it has been shown that this anechoic liquid component is more likely to be pure sebum that becomes liquid at body temperature [6]. Additionally, a study demonstrated that 3D ultrasound has more benefits for the physician, compared to 2D ultrasound. Therefore, lesions appear in 3D ultrasound as spherical, globular structures, in a larger number and some adherent to each other, characteristics similar to those described macroscopically after laparotomy [7]. Further, we will proceed to describe mature ovarian teratomas with intra-cystic spherical ‘balls’, which are present in our case. There are few reported cases of mature cystic teratomas with multiple floating spherical masses [6]. These have been found in different locations, such as the ovary, retroperitoneum [8], and mediastinum [9], all of them with different compositions based on the mentioned region. According to Kawamoto et al., the appearance of multiple floating spheres within a pelvic cystic tumor has not been described in other pelvic lesions, making it a pathognomonic element for mature cystic ovarian teratomas [10]. The presence of the floating balls sign is more common in larger teratomas with a thicker wall, as they require more space to form [3]. It is speculated that the formation of spherical masses occurs through the aggregation of sebum around a nidus, composed of small debris, desquamated material, or fine hair strands. Aggregation is also slightly facilitated by the peristalsis of the small intestine, which is in contact with the cyst wall, but it takes a long time for them to form, explaining the slow growth rate of the lesions, at approximately 1.8 mm/year [3,10]. However, there is a case described by Donnadieu et al. [11] where a pregnant patient had a follow-up ultrasound at 22 weeks of gestation, which revealed an ovarian cystic mass with spherical lesions inside that continued to increase in size up to 20 cm within a few weeks. This case was considered the first case of mature cystic ovarian teratoma with spherical lesions in pregnant women.

Due to its ability to distinguish different tissue types by their densities, even in very small amounts, CT imaging has a higher sensitivity for diagnosis compared to ultrasound but is less recommended due to ionizing radiation risk. This type of ovarian teratoma exhibits distinct imaging characteristics, which include the presence of low-density spherical masses containing a mix of fat, debris, hair strands, and fluid. Calcifications or the dermoid plug formed from the cyst wall can also be observed. In the case of a ruptured cyst, these hypodense masses are randomly distributed in the abdomen and pelvis, predominantly in the pelvic area, surrounded by the leaked intracystic fluid. In case of a ruptured cyst, the literature emphasizes that the fluid distributes in antidependent pockets, and may lead to chemical peritonitis. This may affect the mesentery and thickening of the peritoneum, closely mimicking peritoneal carcinomatosis [1,35], as initially suspected in our case through the CT examination. Malignant transformation can be suggested by the enlargement of the cystic ovarian teratoma >10 cm, soft tissue plugs, and the irregular/crenelated appearance of the cyst’s wall. Although the presented teratoma was approximately 14 cm in the largest diameter, malignancy was excluded after histopathological examination. MRI is the investigation reserved for difficult-to-diagnose cases; it is extremely sensitive to fatty structures, and contrast can identify invasive solid components, making it necessary for differentiating malignant elements. It is the preferred investigation for women of childbearing age. On T1-weighted sequences, the spherical masses have a hyperintense periphery compared to the intracystic fluid, which has a hypointense center. In contrast, on T2-weighted sequences, the signal is opposed: hypointense at the periphery and hyperintense in the center. The core section consists predominantly of hair strands and soft tissues, while the main component of the globular masses is represented by fat/sebum, which appears suppressed on fat saturation sequences [7]. A characteristic sign of these lesions is the “boba sign” [36], inspired by a Taiwanese drink called bubble tea, which contains multiple tapioca pearls, having an imaging ap-pearance similar to the globular masses in mature cystic ovarian teratoma. These MRI characteristics of the globular masses were observed in our patient as well, noting that the lesions and intracystic fluid were dispersed throughout the abdomen and pelvis, indicating a complicated ruptured teratoma. In case of a suspected teratoma, the most important MRI sequence is represented by “chemical shift” imaging. Due to the fact that all spheres contain a large amount of fat, in the opposed phase images these spheres present a homogeneous signal drop-out, which makes the diagnosis straight-forward. To our knowledge, this is the first reported case of a ruptured cystic teratoma with ‘floating balls’ that has both CT and MRI imaging, making this case report an extremely useful teaching material.

## 5. Conclusions

In conclusion, we can highlight the fact that mature cystic ovarian teratoma is, in most cases, an asymptomatic tumor discovered incidentally. Cystic ovarian teratomas manifest in various forms, and the presence of floating balls is rare. However, what has not yet been previously reported in the literature is the situation where a mature cystic ovarian teratoma with floating globules complicates through rupture, making the diagnosis easily to be confused with peritoneal carcinomatosis. Therefore, awareness of this possibility is crucial.

## Figures and Tables

**Table 1:** Complete blood count. This table displays various laboratory tests along with their conventional units, observed values, and reference range values.

Laboratory Test	Conventional Units	Value	Reference Range Value
WBC (White Blood Cells)	$\times 10^{3}/\mu$L	8.2	4–9.5
Erythrocytes	$\times 10^{3}/\mu$L	4.49	4–5.5
Hemoglobin	g/dL	13.12	11.5–15
Thrombocytes	$\times 10^{3}/\mu$L	252	150–400
Creatinine	g/dL	0.8	0.7–1.2
Blood Glucose	g/dL	106	75–110
ALT (Alanine Aminotransferase)	U/L	24	0–35
AST (Aspartate Aminotransferase)	U/L	24	14–36

WBC, white blood cells; AST, aspartate aminotransferase; ALT, alanine aminotransferase.

**Table 2:** Comparison of Imaging Modalities for Ovarian Teratomas and Peritoneal Carcinomatosis.

Imaging Technique	Ovarian Teratoma Findings	Peritoneal Carcinomatosis Findings
Ultrasound (USG)	Echogenic mass characteristics include diffusely or partially echogenic mass with posterior sound attenuation due to sebaceous material and hair, echogenic interface at the edge (“tip of the iceberg” sign), and mural hyperechoic Rokitansky nodule (dermoid plug). Observable features include echogenic, shadowing calcific or dental components, fluid-fluid levels, and multiple thin, echogenic bands (“dot-dash pattern”). Color Doppler imaging reveals no internal vascularity, indicating the need for further investigation to rule out malignancy. The “intracystic floating balls” sign is rare but indicative [12,13,14,15,16,17,18,19].	Detection of peritoneal fluid and assessment of its volume. Characterization of ascites as corpuscular and septate, indicating malignancy. Identification of nodules and sheet-like tumor masses as direct signs of peritoneal metastasis. Observation of mesentery and omentum thickening, adhesions, and impaired peristalsis. Tumor deposits detection in the periumbilical region (Sister Mary Joseph nodules). Preoperative assessment of tumor extension for surgical planning. High accuracy in staging advanced ovarian cancer [20].
Computed Tomography (CT)	CT’s high sensitivity for diagnosing cystic teratomas is noted, though its routine use is limited by ionizing radiation exposure. CT findings include: (a) Fat with very low Hounsfield values, (b) Fat-fluid level, (c) Calcification (sometimes dentiform), (d) Rokitansky protuberance, (e) Tufts of hair, diagnostic in 98% of ovarian cystic teratomas. Malignant transformation is suspected with size >10 cm or presence of soft tissue plugs and cauliflower appearance with irregular borders. Ruptured cysts leading to hypoattenuating fatty fluid below the right hemidiaphragm and escaped cyst content causing chemical peritonitis, mesentery stranding, and peritoneum thickening may mimic peritoneal carcinomatosis [14,21,22,23,24,25,26].	Observations in the peritoneal cavity include multifocal discrete nodules to infiltrative masses, omental haziness, ascites, and peritoneal thickening, nodularity, and enhancement. Intraperitoneal seeding is influenced by the flow of peritoneal fluid, determined by ligaments and mesentery attached to the peritoneum. Common locations for peritoneal seeding: (a) Peritoneal reflection of the pelvis, (b) Lower small bowel mesentery, (c) Sigmoid mesocolon, (d) Right paracolic gutter, (e) Right subphrenic space [27].
Magnetic Resonance Imaging (MRI)	MRI evaluation is typically reserved for challenging cases. Highly sensitive to fat components, utilizing both fat suppression techniques and chemical shift artifact to confirm fat presence. Capable of identifying solid invasive components, aiding in the accurate local staging of malignant variants. Offers diffusion-weighted imaging, detailed morphological and functional characteristics, high tissue contrast resolution, superior soft tissue contrast, and good tissue characterization [1,28,29].	Increased enhancement of peritoneal metastases, often greater than liver enhancement, best seen after 5–10 min [30]. Combination of morphologic sequences with diffusion sequencing demonstrates a 20% increase in sensitivity by MRI in the identification of lesions [31,32,33]. MRI shows high sensitivity, with a sensitivity of 85–90% [34].

## Data Availability

Data are available upon request to the corresponding author.
